# Exploring the impact of cuproptosis on prostate cancer prognosis *via* RNA methylation regulation based on single cell and bulk RNA sequencing data

**DOI:** 10.3389/fphar.2025.1573611

**Published:** 2025-04-01

**Authors:** Junchao Wu, Wentian Wu, Jiaxuan Qin, Ziqi Chen, Rongfang Zhong, Peng Guo, Song Fan

**Affiliations:** ^1^ Department of Urology, The First Affiliated Hospital of Anhui Medical University, Hefei, China; ^2^ Institute of Urology, Anhui Medical University, Hefei, China; ^3^ Anhui Province Key Laboratory of Urological and Andrological Diseases Research and Medical Transformation, Hefei, China; ^4^ Department of Oncology, The First Affiliated Hospital of Anhui Medical University, Hefei, China; ^5^ Department of Urology, The University of Hong Kong-Shenzhen Hospital, Shenzhen, China; ^6^ Department of Urology, The Affiliated Jiangyin Hospital of Nantong University, Wuxi, China

**Keywords:** prostate cancer, cuproptosis, RNA methylation regulators, immunotherapy, chemotherapy agent

## Abstract

**Background:**

Cuproptosis, along with RNA methylation regulators, has recently come to the fore as innovative mechanisms governing cell death, exerting profound impact on the onset and progression of multiple cancers. Nonetheless, the prognostic implications and underlying regulatory mechanisms of them associated with prostate cancer (PCa) remain to be thoroughly investigated.

**Methods:**

Genomic and clinical data for PCa from The Cancer Genome Atlas datasets were analyzed to identify a prognostic model through univariate and Least Absolute Shrinkage and Selection Operator Cox regression analyses that were validated utilizing external datasets. We used receiver operating characteristic curves and C-index to evaluate the accuracy of our prognostic model. In conjunction with this, we conducted single-cell RNA sequencing (scRNA-seq) analyses to investigate underlying mechanisms and evaluate the degree of immune infiltration, as well as to assess patients’ responses to diverse chemotherapy agents. Especially, qPCR assay was utilized to unveil the expression of signature genes in PCa.

**Results:**

We meticulously selected six Cuproptosis-Associated RNA Methylation Regulators (CARMRs) to establish a risk prognosis model, which was further verified to obtain enhanced predictive capacity in external validation cohorts. Insights from immune infiltration and scRNA-seq analyses have elucidated the immune characteristics of PCa, and highlighted the immunosuppressive role of regulatory T cells on immune response. Additionally, drug susceptibility analysis demonstrated that patients with PCa in the low-risk category derived better benefit from bicalutamide treatment, whereas those in the high-risk group exhibited a favor response to adriamycin and docetaxel treatments. The qPCR and immunohistochemistry (IHC) staining assays also reveal the a dramatically altered expression pattern of TRDMT1 and ALYREF in PCa tissues.

**Conclusion:**

In general, we established a model involving CARMRs that can better predict the risk of recurrence of PCa and have identified the possible mechanisms affecting PCa progression, thereby promoting further research in this field.

## 1 Introduction

Prostate cancer (PCa) is a global health issue. According to the statistics from GLOBOCAN 2020, PCa serves as the second most common cancer in men worldwide following lung cancer, and it is particularly endemic in 112 countries (Sung et al., 2021). Localized PCa is primarily treated through radical prostatectomy combined with radiotherapy, whereas high-risk PCa is managed *via* androgen deprivation therapy (ADT) (Sekhoacha et al., 2022). Nevertheless, long-term ADT treatment may contribute to the occurrence of castration-resistant PCa (CRPC), resulting in a higher risk of metastasis and poorer recurrence-free survival (RFS) (Bach et al., 2014; Teo et al., 2019). Nowadays, clinical diagnostic biomarkers used to screen the public for PCa, such as prostate specific antigen, lack specificity and the Gleason score can be easily affected by sampling error and subjectivity. In contrast, genotyping-based classification can be crucial in identifying specific subtypes of PCa and promoting individualized treatment (Kench et al., 2022; Ye et al., 2020). Therefore, exploring novel biomarkers for predicting the prognosis of PCa and improving treatment accuracy is of great significance.

Cuproptosis, a novel non-apoptotic mode of cell death, is mediated by copper-dependent mitochondria and occurs through direct binding of copper to the acylation components in the tricarboxylic acid cycle. Increased intracellular levels of copper ions can induce cuproptosis (Chen et al., 2022). Recent research has shown that copper levels in tumor tissues are 2–3 times higher than those in normal tissues (Gupte and Mumper, 2009). As a common form of cell death, cuproptosis has the capacity to regulate the DLAT/mTOR pathway to enhance the autophagy of PCa cells and reverse their resistance to chemotherapy drugs (Wen et al., 2023). Given that cuproptosis induces cell death *via* changing mitochondrial metabolism, the drugs that enhance this dependency, such as enzalutamide could build on synergies (Gao et al., 2024). The discovery of cuproptosis, which needs further research, may provide ideas for the exploration of novel therapeutic targets for cancer treatment. Moreover, gaining an insight into the mechanism and associated signaling pathways of cuproptosis would provide new options for reversing drug resistance in PCa.

On the other hand, RNA methylation modifications participate broadly in biological processes and correlate with proliferation, metastasis, cellular stress, and the immune response to cancer (Yang et al., 2021; Chen et al., 2019). The RNA methylation categories of significance included N6-methyladenosine (m6A), 5-methylcytosine (m5C), N7-methylguanosine (m7G), and N1-methyladenosine (m1A) (Long et al., 2023). Among these, the m6A modification is the most widely distributed in living organisms and is known to be involved in multiple processes of RNA synthesis (An and Duan, 2022). For example, YTHDF2 has been shown to induce the proliferation of PCa cells through an m6A--dependent mechanism (Li et al., 2020). Elevated methylation levels of EI3C mRNA may contribute to metastasis by activating the MAPK pathway (Ding et al., 2022). Additionally, METTL3 (Haigh et al., 2022), VIRMA (Barros-Silva et al., 2020), FTO (Zhang J. et al., 2023), and RBM15 (Wang et al., 2023) are known to play key roles in controlling the extent of methylation to impact the survival, progression and drug-resistance of patients with PCa by serving as “writers”. RNA methylation has also shown a strong correlation with genetic variation, alternative splicing, and immune phenotypes. We speculate that the tumor microenvironment (TME) would be remodeled when the methylation level increased (Zhao et al., 2021). RNA methylation modifications have been implicated in various cancers making them potential biomarkers for cancer diagnosis and treatment.

To conclude, rational prognostic models were formerly established involving Cuproptosis-Associated RNA Methylation Regulators (CARMRs) to provide fresh insights into the development of new targets and patient immunotherapy in colorectal cancer and hepatocellular carcinoma (Li et al., 2023; Zhao et al., 2024a). CARMRs may help characterize the immune status, be essential for proliferation and invasiveness, and predict patient prognosis. Hence, we constructed a CARMR-based prognostic model to explore the potential mechanism of transfer and drug resistance in PCa.

**FIGURE 1:**
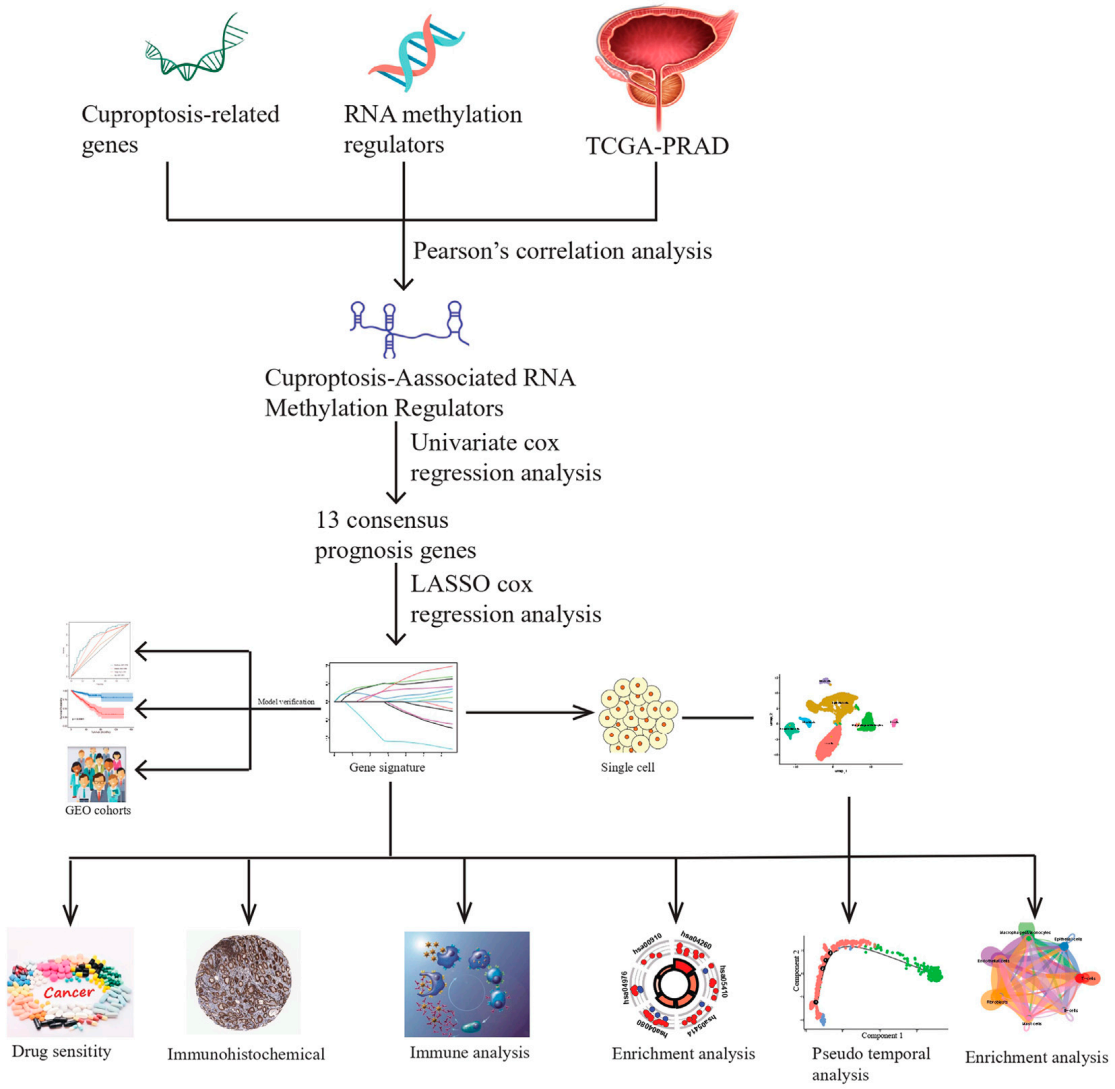
A schematic showing the workflow of the study.

In this study, we constructed a prognostic model utilizing CARMRs in patients with PCa to divide them into high- and low-risk groups, predict RFS, the landscape of TME, and drug sensitivity. We believe that clinicians can better identify the PCa stage and guide individualized treatment through our approach described here. The workflow of this study is shown in Figure 1.

## 2 Materials and methods

### 2.1 Datasets acquisition and preprocessing

Two PCa cohorts were included in this study. The Cancer Genome Atlas Prostate Adenocarcinoma (TCGA-PRAD) cohort was downloaded from the Genomic Data Commons. We deleted patients with missing data, and eventually enrolled 488 patients with complete expression profile data and clinical information. We transformed Transcripts Per Million (TPM) data to a log_2_ (TPM) format to achieve better comparability. Subsequently, the Gene Expression Omnibus (GEO) cohorts from three eligible GEO datasets, GSE21032 (n = 138) (Taylor et al., 2010), GSE116918 (n = 223) (Jain et al., 2018), and GSE46602 (n = 36) (Mortensen et al., 2015) were utilized as validation cohorts. The baseline information of all cohorts above has been summarized in Table 1. We removed potential cross-dataset batch effects using the “sva” library of R software package implemented *via* an empirical Bayes framework (Leek et al., 2012). A single-cell dataset (GSE193337) (Heidegger et al., 2022) was extracted from the GEO database. The baseline information of samples from the single-cell dataset was summarized in Table 2 and the single-cell RNA sequencing (scRNA-seq) data of four PCa samples were utilized for our study.

**TABLE 1:** Summary of the clinicopathological parameters of the four enrolled datasets.

Items	TCGA-PRAD (n = 488)	MSKCC(n = 138)	GEO116918 (n = 223)	GEO46602 (n = 36)
Age				
≤60	219	87	31	15
>60	269	51	192	21
Pathological T stage				
T1 + T2	187	86	127	19
T3 + T4	301	52	96	17
Gleason score				
≤7	287	117	127	32
>7	201	21	96	4
Status				
Recurrence free	396	103	172	14
Recurred	92	35	51	22

**TABLE 2:** After filtering, basic quality control statistics for the respective combined datasets, and patient samples are characterized in the table below.

Dataset	Sample	Number of cells	Number of genes
GSE193337	GSM5793828	2,420	19,815
	GSM5793829	5,724	21,344
	GSM5793831	5,087	21,890
	GSM5793832	5,964	21,530

### 2.2 Identification of prognostic CARMRs

We identified 13 cuproptosis-related genes and 59 RNA methylation regulators, which were treated as the focus of our study based on previous literature (Chen YS. et al., 2021; He et al., 2022; Li M. et al., 2022; Tsvetkov et al., 2022). A Pearson correlation analysis was employed to identify CARMRs, and filter conditions were set to |R|≥ 0.4,*P* < 0.001. Further, we conducted a univariate Cox regression analysis utilizing “survival” package (Liu et al., 2021) and *P* < 0.05 was designated as a threshold to identify CARMRs that were correlated with RFS.

### 2.3 Construction and validation of a prognostic model and nomogram

Based on the results of our univariable Cox regression analysis, we used least absolute shrinkage and selection operator regression analysis (LASSO) regression analysis (Li Y. et al., 2022) to select combinations of genes that were rationally narrowed by the glmnet software package, in order to minimize the risk of overfitting. Through cross-verification, we selected the penalty parameter (λ) value with the least average error to construct the model. Finally, we selected six prognostic CARMRs to construct a risk model, and calculated the risk scores for each patient with PCa according to the following equation:

$$\text{Risk score}=\sum_{i=1}^{n}\left({\text{coef}}_{i}\;*\exp_{i}\right)$$

where coef_i_ and exp_i_ terms represent the coefficients and expression values of the prognostic genes, respectively. Defining the median risk score of the TCGA cohort as the cutoff value, the patients of the TCGA and GEO cohort could be separated into high- and low-risk groups. Time-dependent receiver operating characteristic (ROC) curves and Kaplan-Meier (KM) curves were used to evaluate the predictive performance of our prognostic model in the TCGA-PRAD and GEO cohorts. Combining clinicopathological factors with prognostic significance, we constructed a nomogram for the TCGA-PRAD cohort *via* the “regplot” package to predict 1-, 3- and 5-year risk of recurrence for patients with PCa. We then computed the C-index value to show the predictive performance of our nomogram and other clinicopathological parameters. The calibration curve also evaluated the efficacy of the nomogram. Finally, we analyzed the relationship between different independent factors and risk scores and plotted the KM curves in the clinicopathological subgroup to further verify the capability of this approach.

### 2.4 Function enrichment analysis

The differentially expressed genes (DEGs) of the high-risk and low-risk group patients with PCa were determined using the “DESeq2” package with the threshold of |log_2_ foldChange| ≥ 0.5 and adjusted *P* < 0.05. We then investigated biological structure and function, using gene ontology (GO) enrichment and Kyoto Encyclopedia of Genes and Genome (KEGG) enrichment analyses to identify pathways enriched in PCa. The changes in signaling pathways and interactions of DEGs were depicted by Gene Set Enrichment Analysis (GSEA). Various software packages, namely “clusterProfiler” (Wu et al., 2021), “org.Hs.e.g.db” (Qing et al., 2022) and the R software package, were employed.

### 2.5 Drug sensitivity and immune infiltration analysis

Understanding the responsiveness of patients carrying various levels of risk of PCa recurrence due to the administration of common chemotherapeutic agents contributes to developing individualized treatment plans for patients with PCa. Therefore, we used the “pRRophetic” software package to calculate the half maximum inhibitory concentration (IC_50_) value of adriamycin, bicalutamide and docetaxel in different risk of PCa recurrence subgroups. In order to explore the relationship between risk score and TME, the CIBERSORT (Craven et al., 2021) algorithm was utilized to investigate the differential proportions of 22 kinds of immune cells between high- and low-risk groups in patients with PCa. The ESTIMATE algorithm (Xu et al., 2021) was utilized to calculate estimate score, immune score, stromal score and tumor purity. We utilized the ssGSEA algorithm (Ye et al., 2019) to validate the accuracy of immune infiltration analysis. Finally, we analyzed the expression of TRDMT1 and ALYREF in tumor and normal histopathology tissue sections acquired from the Human Protein Atlas database (www.proteinatlas.org).

### 2.6 Quality control and annotation of single-cell RNA sequencing data

The “Seurat_v5” software package was used to further process single-cell RNA sequencing data derived from PCa samples (Yu et al., 2022). First, we constructed single cell objects by the CreateSeuratObject method. With the threshold of RNA features ranging from 300 to 7,000 and the proportion of a mitochondrial gene set to less than 5%, eligible cells were selected and retained for further analysis. We employed the “NormalizeData” algorithm to standardize the data and selected the top 20 components and first 2,000 variably expressed genes for follow-up analysis, while “ScaleData” was used to center and scale the highly variable genes. We conducted principal component analysis (PCA) to reduce the dimensionality of the data, and the PCA number was adjusted to 15. The “Harmony” algorithm was applied to integrated single-cell data from different datasets to eliminate potential batch effects. Subsequently, we defined a category of genes with the same expression patten as a cluster and utilized the software function of uniform manifold approximation and projection (UMAP) to depict the distribution of each cluster. Cell annotation was performed by artificiality, and the cell markers were referred from CellMarker2.0.

### 2.7 Cell-cell communication and pseudotime analysis

We applied “Cellchat” from R software package to reveal and visualize the cell-cell interactions and possible signaling pathways involved (Yu et al., 2022). After identifying the prognostic genes primarily distributed in T cells, we subdivided and annotated the T cell cluster on the basis of cell markers obtained from previous studies (resolution = 0.5). We investigated the transition of different subtypes utilizing the “Monocle” application from R software package. The resulting cell state plots and cell type maps revealed the developmental trajectory of PCa.

### 2.8 Quantitative real-time PCR (qRT-PCR)

The RNA extraction was performed using the Trizol reagent (Beijing ComWin Biotech Co., Ltd.) from prostate normal and cancer cell lines RWPE-1, LNCaP, C42, PC3 (Wuhan Pricella Biotech Co., Ltd.). For cDNA synthesis, reverse transcription was conducted using the TaKaRa (Dalian TaKaRa Biotech Co., Ltd.) kit according to the manufacturer’s instructions. GAP was employed as an internal reference gene to normalize relative expressions of lncRNA with the 2^−ΔΔCT^ method. The specific primers in our study were as follows: TRDMT1 (forward: 5′-CGG​GTG​CTG​GAG​CTA​TAC​AG-3′, reverse:5′-CGACAGTGTTGACATCAATGGC-3′); ALYREF (5′-GCA​GGC​CAA​AAC​AAC​TTC​CC -3′, 5′-AGT​TCC​TGA​ATA​TCG​GCG​TCT -3′).

### 2.9 Statistical analysis

All statistical analyses were carried out using R software package (version 4.2.0). The continuous data were analyzed by independent t-test, Wilcoxon test or Fisher’s exact test, which was considered to analyze classified data. *P* < 0.05 was considered to be statistically significant.

## 3 Results

### 3.1 Identification of cuproptosis-associated RNA methylation regulators (CARMRs) and construction of a prognostic model involving CARMRs

**FIGURE 2:**
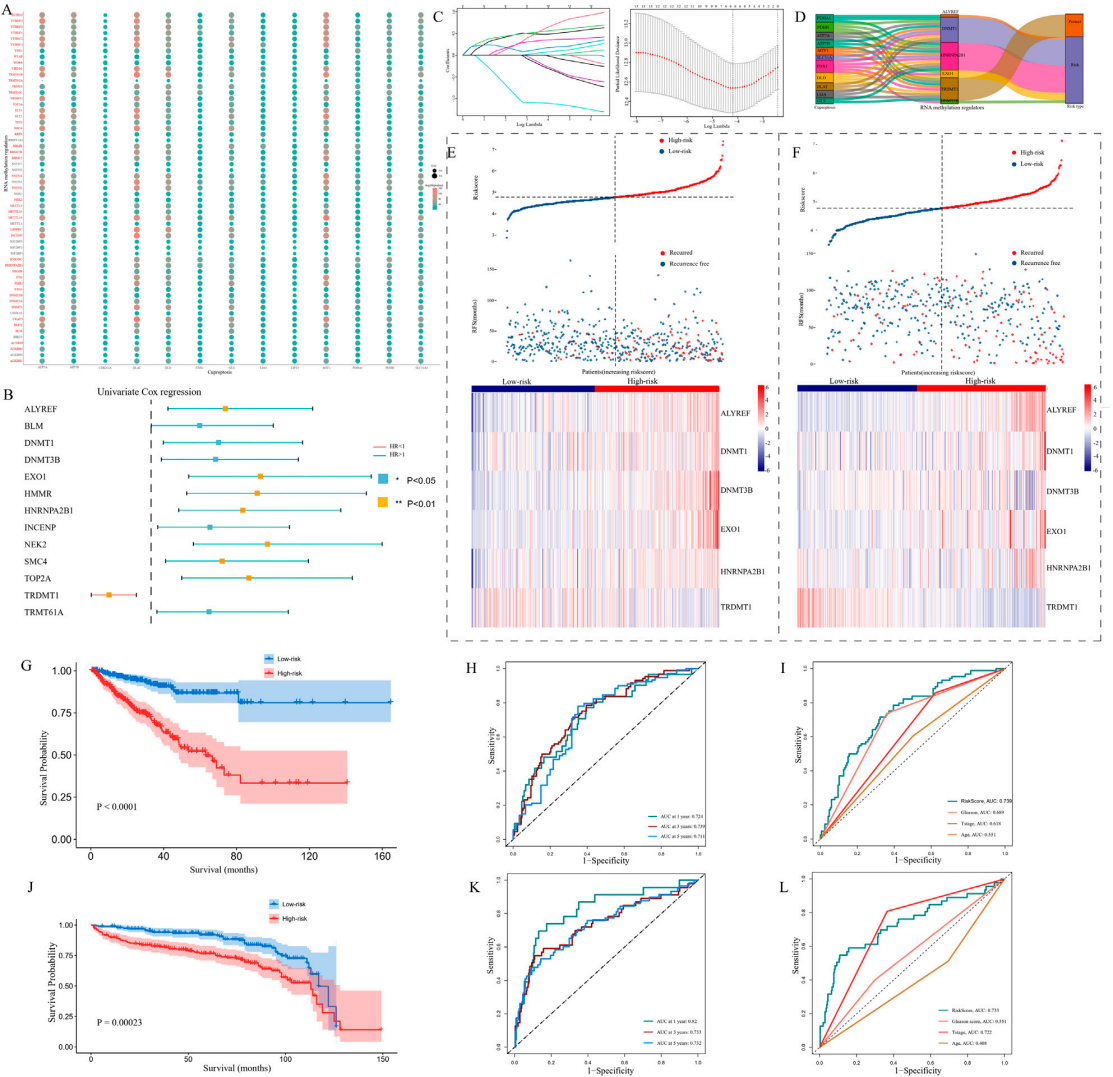
The construction and validation of a prognostic model. **(A)** An association between 13 copper death-related genes and 56 RNA methylation regulatory genes is presented by a dot plot. **(B)** Univariate Cox regression analysis results showing that Cuproptosis-Associated RNA Methylation Regulators (CARMRs) were associated with the prognosis of patients with PCa. **(C)** Selection of prognostic CARMRs on the basis of the optimal λ calculated by LASSO regression analysis. **(D)** Sankey diagram revealing the correlation between cuproptosis-related genes and RNA methylation regulatory genes. **(E,F)** Risk maps depicting the distribution of patient status and the expression profiles of prognostic genes in TCGA and GEO cohorts. **(G–I)** KM curve, Time-independent ROC curve, and clinical ROC curve in a TCGA cohort. **(J–L)** Validation of our prognostic model by conducting KM curve, time ROC curve, and clinical ROC curve analyses in a GEO cohort. *P < 0.05,**P < 0.01,***P < 0.001.

We conducted Pearson’s correlation analysis between cuproptosis-related genes and RNA methylation regulators: by setting filter criteria, a total of 48 CARMRs were selected for further study (Figure 2A). To screen out CARMRs correlated with RFS, we performed univariate Cox regression analysis and identified 13 CARMRs significantly associated with prognosis (*P* < 0.05) (Figure 2B). The results revealed that only TRDMT1 exhibited a protective effect, whereas the remaining 12 genes (NEK2, SMCA, ALYREF, BLM, DNMT1, DNMT3B, TOP2A, TRMT61A, HMMR, EXO1, HNRNPA2B1, INCENP) were identified as risk factors for the recurrence of PCa. Further, we conducted LASSO Cox regression analysis to minimize model overfitting and determine the optimal λ value (Figure 2C). Ultimately, a six-CARMR signature, comprising a set of genes, was selected for the construction of a prognostic model assessing the risk of recurrence within PCa and the association between cuproptosis-related genes and the six select RNA methylation regulators was vividly visualized using a Sankey diagram (Figure 2D). Risk scores were calculated as follows: Risk Score = (0.458731758 × ALYREF) + (0.208975015 × DNMT1) + (0.462010845 × DNMT3B) + (0.136363177 × EXO1) + (0.219757226 × HNRNPA2B1) + (−0.864908568 × TRDMT1), and the gene coefficient was derived from the results of the LASSO regression analysis. Setting the median risk score of the TCGA cohort as the cutoff value based on equations, all patients could be separated into high- and low-risk PCa recurrence groups. The risk factor association plot depicted the distribution of risk scores, PCa recurrence status, and expression levels of the model genes among patients in the high- and low-risk groups, respectively (Figure 2E). The KM curves revealed that the high-risk group had a significantly worse prognosis than that of the low-risk group (*P* < 0.001) (Figure 2G). In order to measure the efficacy of our prognostic model, ROC curves were employed to evaluate the sensitivity and specificity of the model. The results demonstrated that the area under the curve (AUC) for predicting 1-, 3-, and 5-year RFS were 0.724, 0.739, and 0.711, respectively and the predictive performance of the risk scores was better than other clinicopathological parameters (Figures 2H,I), indicating that the constructed model possessed a favorable predictive capability. To further validate our prognostic model, we applied the approaches mentioned above to the GEO cohort data. After separating patients into diverse risk groups, we discovered that the expression levels of the prognostic genes varied among patients and recurred patients were concentrated in the high-risk group (Figure 2F). The KM curve revealed a satisfactory separation of patients (*P* < 0.001) (Figure 2J). Complementary to this, AUC values of ROC curves were all over 0.700, indicating the superior predictive performance of our prognostic model, and the clinical ROC curve confirmed the reliable predictive capability of the computed risk scores (Figures 2K,L).

### 3.2 Nomogram based on independent prognostic factors in patients with PCa and correlation between the CARMR signature genes and clinicopathological traits

**FIGURE 3:**
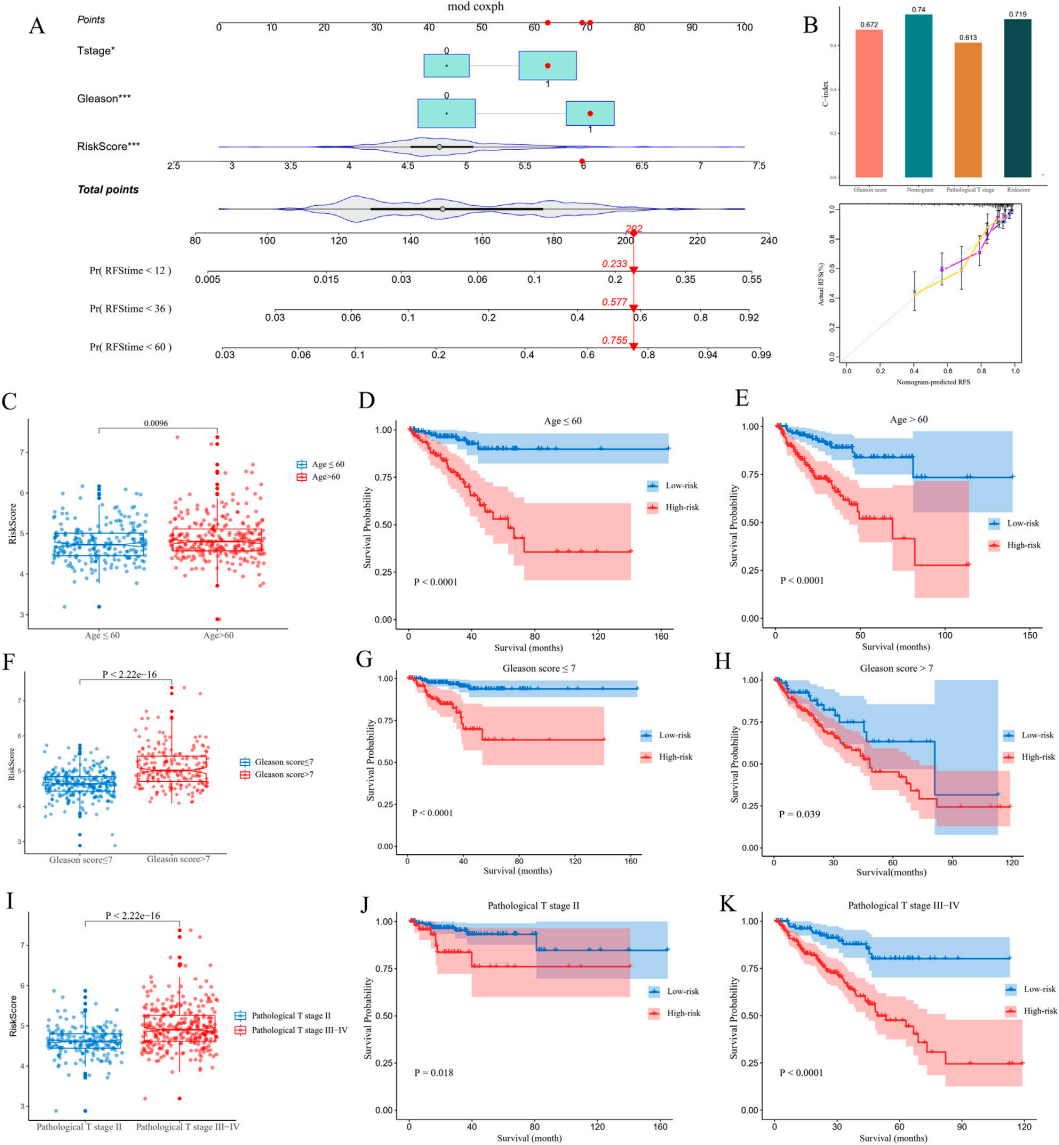
Construction of a nomogram and conducting clinicopathological subgroup analysis. **(A)** A nomogram was utilized to predict 1-, 3- and 5-year recurrence-free survival (RFS) values for patients with PCa in a TCGA cohort. **(B)** C-index, calibration curve of nomogram, and other clinicopathological factors were utilized to evaluate the accuracy of the nomogram. **(C,F and I)** Comparisons of risk score differences between various clinicopathological factor subgroups. **(D,E,G,H,J and K)** KM analysis was conducted to identify survival differences between high- and low-risk groups of patients with PCa in different subgroups.

The nomogram integrated independent prognostic factors which were filtered utilizing a univariate Cox regression analysis, and multiple line segments were displayed in a specific proportion to quantify the 1-, 3-and 5-year probability of PCa recurrence in patients with PCa (Figure 3A). To evaluate the discriminatory ability of the model, we calculated the C-index for the Gleason score, nomogram, pathological T stage and risk score, which were 0.672, 0.740, 0.613 and 0.719, respectively. These results were displayed as a bar plot and indicated that the nomogram had the highest accuracy in predicting PCa recurrence compared with a single norm (Figure 3B). Additionally, calibration curves demonstrated good consistency between the actual and the predicted RFS. To further analyze the relationship between the recurrence risk score and clinicopathological parameters, we grouped the patients in the TCGA-PRAD cohort according to age, Gleason score, and pathological T stage score (Figures 3C,F,I). The results demonstrated that age, Gleason score and T stage score were positively correlated with the risk of PCa recurrence score (*P* < 0.05). KM curves indicated that the CARMR signature set of genes had predictive value for RFS in different stratified cohorts (*P* < 0.05) (Figures 3D,E,G,H,J,K).

### 3.3 Functional enrichment analysis and drug sensitivity analysis

**FIGURE 4:**
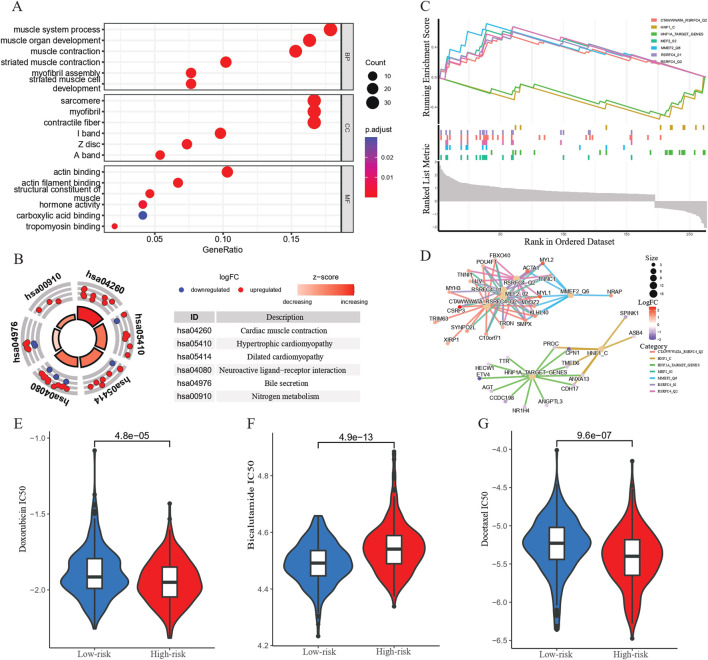
Functional enrichment analysis and chemotherapeutic response prediction of prognostic genes. **(A)** GO enrichment analysis of CARMRs. **(B)** KEGG enrichment analysis of CARMRs. **(C)** GSEA of CARMRs. **(D)** The interaction of genes in different enriched pathways. **(E–G)** Predicting the IC_50_ value in high- and low-risk groups of patients with PCa to doxorubicin, bicalutamide and docetaxel therapy.

An enrichment analysis was conducted on the DEGs between the high- and low-risk groups. The GO analysis results revealed that the enriched biological processes included muscle system processes, muscle organ development, muscle contraction, striated muscle contraction, assembly of muscle fibers, and striated muscle cell development. The enriched cellular components included sarcomeres, muscle fibers, contractile fibers, M-lines, Z-discs, and A-bands. The molecular functions enriched included actin binding, actin filament binding, muscle structural constituent, hormone activity, carboxylic acid binding, and myosin binding (Figure 4A). KEGG analysis demonstrated that the CARMR signature set of genes was involved in pathways related to cardiac muscle contraction, hypertrophic cardiomyopathy, dilated cardiomyopathy, neuroactive ligand-receptor interaction, bile secretion, nitrogen metabolism, and adrenergic signaling in cardiac myocytes (Figure 4B). The GSEA profile showed that the HNF1_C and HNF1A_TARGET_GENES pathways were downgraded, while other pathways were upgraded (Figure 4C). In addition, the gene interaction network diagram revealed promising interactions among the DEGs enriched in various pathways (Figure 4D).

In order to gain a deeper understanding of the drug response, we performed a drug sensitivity analysis of the high- and low-risk groups of patients with PCa. By comparing the IC_50_ values of chemotherapy drugs for treating PCa, we found that the high-risk group had a higher IC_50_ value for bicalutamide than the low-risk group (*P* < 0.001), whereas the IC_50_ values for doxorubicin and docetaxel were lower for the high-risk group as shown in the boxplot, which implies that the high-risk group showed a higher sensitivity to doxorubicin (*P* < 0.001) and docetaxel (*P* < 0.001) compared to the low-risk group (Figures 4E–G). These results may be used to guide personalized therapy.

### 3.4 Immune cell infiltration and correlation analysis

**FIGURE 5:**
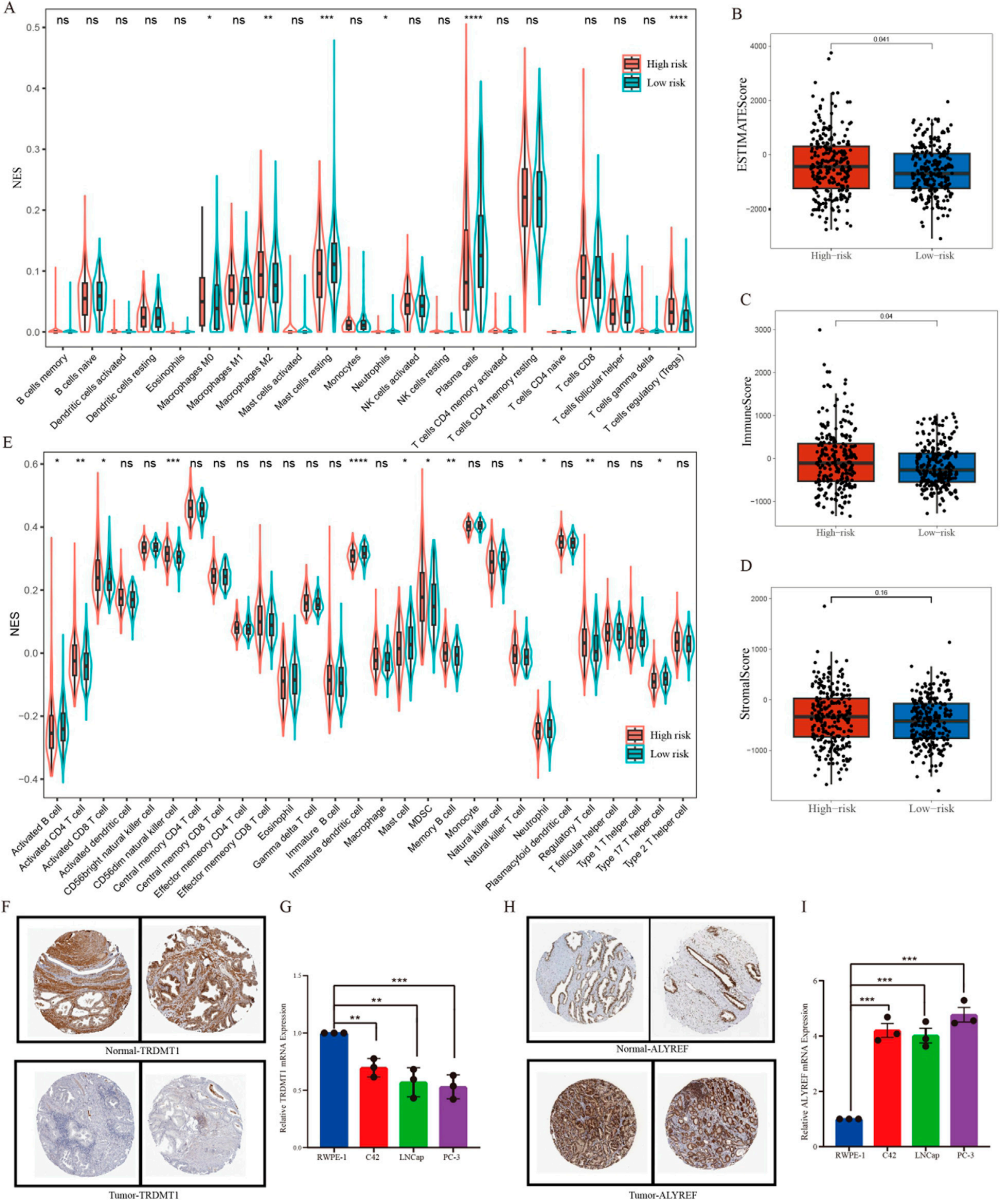
Immune cell infiltration analysis. **(A)** The differences in the enrichment scores of 22 types of immune cells between two risk subgroups. **(B–D)** Comparisons of the differences between two risk subgroups in terms of estimate score, immune score, and stromal score. **(E)** A violin chart showing the differences in the proportions of 28 immune cells between high-risk and low-risk groups of patients with prostate cancer. **(F)** Representative pictures showing the different protein levels of TRDMT1 from HPA. **(G)** Expression of TRDMT1 in normal prostate cell and prostate cancer cells. **(H)** Representative pictures showing the different protein levels of ALYREF from HPA. **(I)** Expression of ALYREF in normal prostate cell and prostate cancer cells. *P < 0.05,**P < 0.01,***P < 0.001.

A violin plot was generated utilizing the Cibersort algorithm to illustrate the proportions of immune cell infiltration in patients with PCa, and the Wilcoxon test was employed to assess the differences in immune cell populations between the high- and low-risk groups. The types of immune cells exhibiting significant differences between the two groups included plasma cells, T regulatory cells (Tregs), M0 macrophages, M2 macrophages, and resting Mast cells (Figure 5A). Further, the results demonstrated that estimate score (Figure 5B), immune score (Figure 5C) and stromal score (Figure 5D) values were elevated in the high-risk group compared with those in the low-risk group (*P* < 0.05). However, there was no difference between the two risk groups in terms of tumor purity. In addition, utilizing the ssGSEA method, we found that the high-risk group exhibited higher levels of activated CD4^+^ T cells, activated CD8^+^ T cells, CD56dim natural killer cells, myeloid-derived suppressor cells (MDSC), memory B cells, nature killer cells and regulatory T cells, whereas it showed a reduction in the levels of activated B cells, immature dendritic cells, mast cells, neutrophils and Type 17 T helper cells (Figure 5E). To delineate the expression profiles of signature genes, we initially investigated the IHC results obtained from the Human Protein Atlas (HPA) dataset, and ascertained the downregulated expression of TRDMT1 and the upregulated expression ALYREF in PCa tissues compared to normal tissues (Figures 5F,H). Furthermore, qPCR assay was employed across a range of diverse cell lines and also demonstrated the decreasing expression trend for TRDMT1 from normal to malignant cells (Figure 5G). Conversely, the expression level of ALYREF exhibited an opposite trend (Figure 5I).

### 3.5 Distribution of prognostic genes in single-cell landscape

**FIGURE 6:**
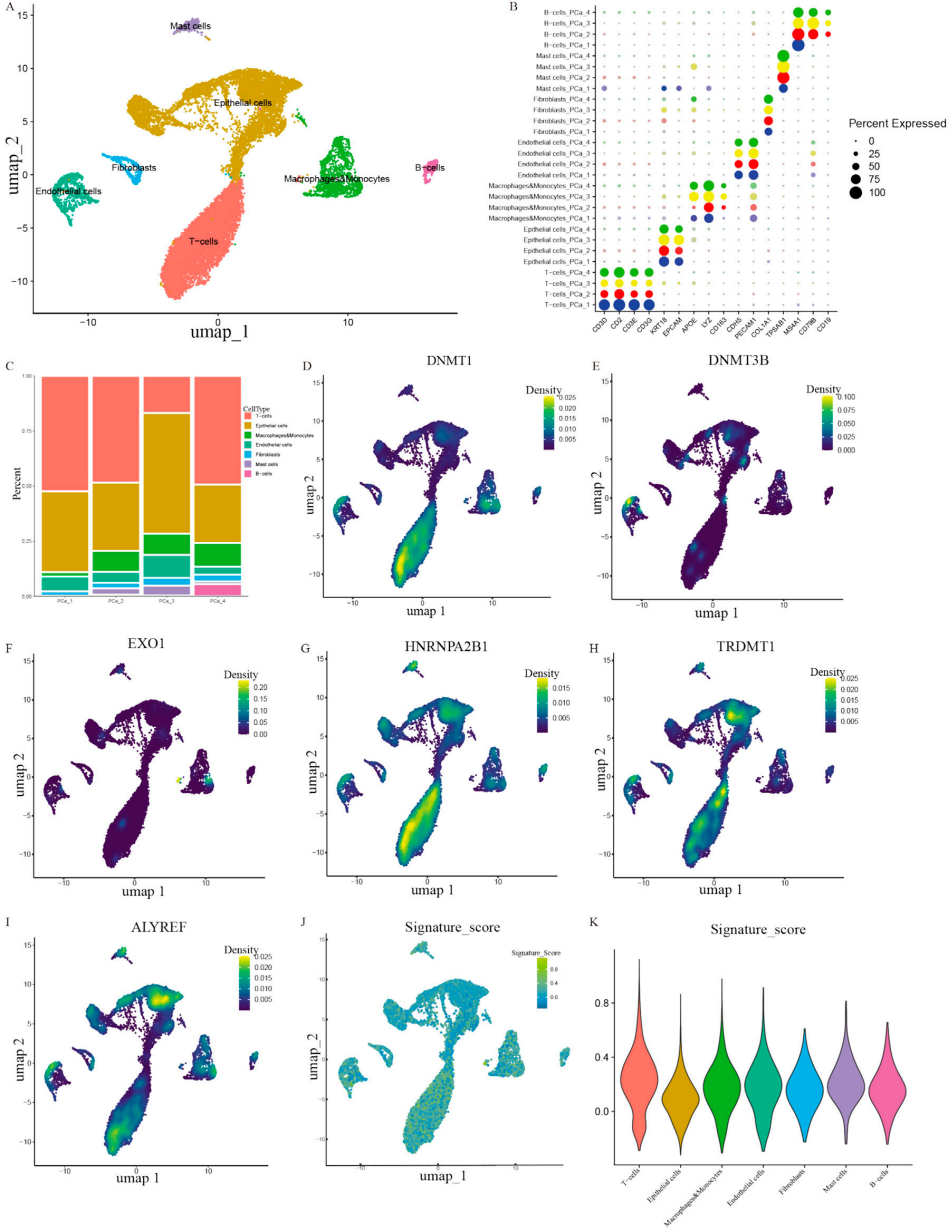
Exploring the distribution of prognostic genes. **(A)** A plot of the different types of cells. **(B)** A bubble plot of the expression of diagnostic marker genes in each cell cluster. **(C)** Cell proportions in four PCa samples. **(D–I)** The distribution of prognostic genes in different clusters. **(J–K)** UMAP map and violin plot indicating the enrichment of gene signatures in PCa tissues.

To further explore the expression profiles of prognostic genes in depth, we probed gene expression in PCa tissue at the single-cell level from a GEO dataset (GSE193337). We extracted the scRNA-seq data of four samples from patients with PCa. The baseline characteristics of patients have been described previously (Heidegger et al., 2022). Utilizing stringent data quality control, we extracted 23,697 cells and 17,618 genes to construct a PCa single-cell atlas. To eliminate the batch effect between distinct samples, we applied harmony function and further establish an initial diagram of PCa tissues. The plots before and after batch effect removal were depicted in Supplementary Figures S1A and B. The Seurat software package was employed to reduce the dimensionality of data. We successfully classified the cells into 14 clusters. We utilized recognized cell markers (Heidegger et al., 2022; Kfoury et al., 2021; Ma et al., 2020; Zaidi et al., 2024), and all clusters were annotated as B cells, epithelial cells, endothelial cells, fibroblasts, mast cells, macrophages, monocytes, and T cells (Figures 6A,B). Notably, we discovered that epithelial cells and T cells were the top two abundant cells in the four samples (as shown in the histogram), implying that these cell types figured prominently in the development of PCa (Figure 6C). We investigated the distribution of prognostic genes in all cell types, and discovered that DNMT1, EXO1 and HNRNPA2B1 were primarily distributed in T cells, whereas DNMT3B was detected in endothelial cells (Figures 6D–G). TRDMT1 and ALYREF were both expressed by T cells and epithelial cells (Figures 6H,I). To identify the cell clusters enriched in prognostic genes, we scored individual cells for their prognostic gene signature and found that the prognostic genes were strongly enriched in a subtype of cells within the T-cell cluster compared with other cell types utilizing ANOVA analysis (*P* < 0.001) (Figures 6J,K).

### 3.6 Intracellular interactions, pseudotime, and function enrichment analysis revealing the role of T cells

**FIGURE 7:**
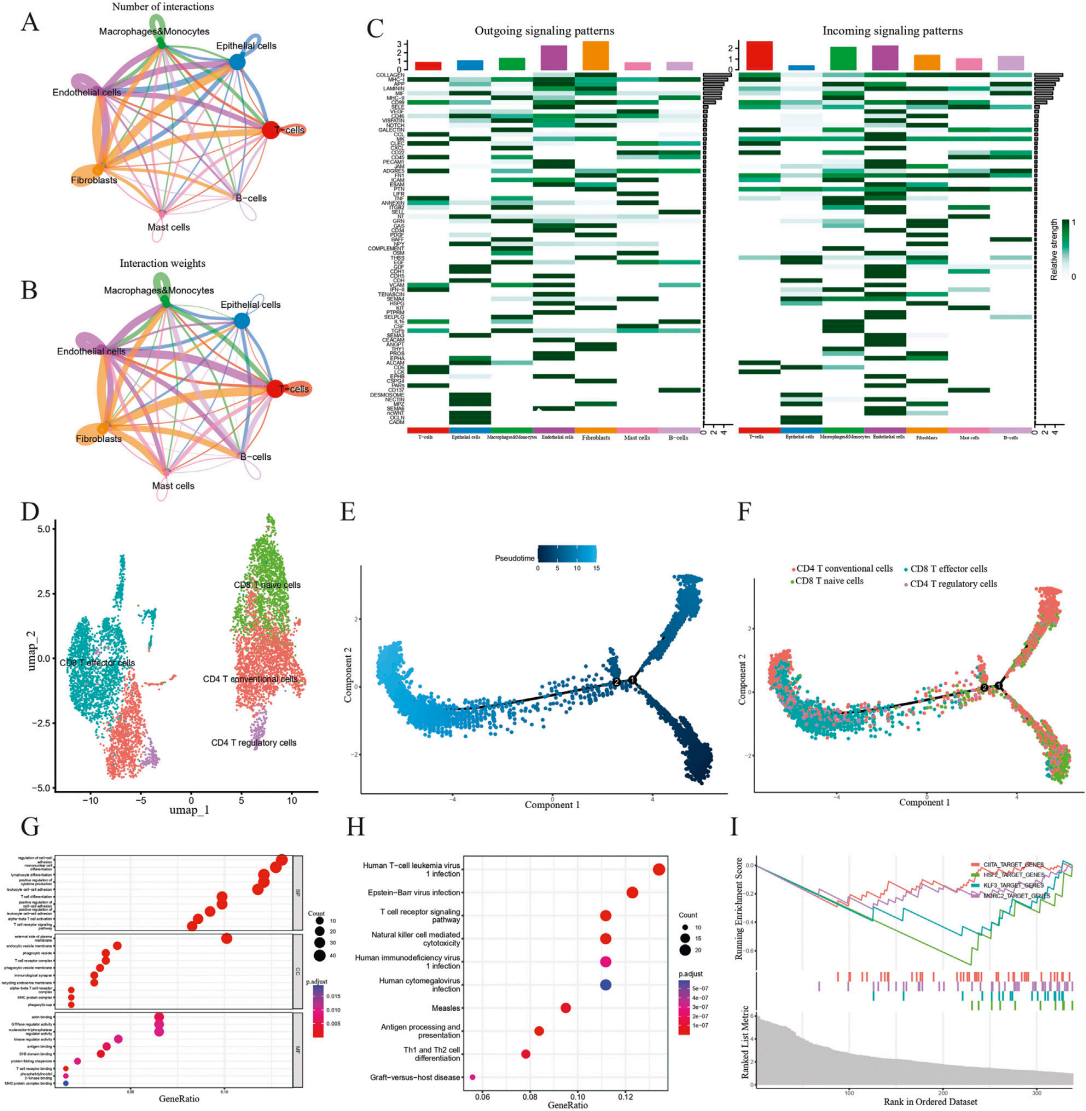
Investigating the role of T cells in the development of PCa. **(A and B)** Cell-cell communication analysis in terms of weighted interactions. **(C)** A heatmap showing signaling pathways in seven clusters. **(D)** Subgroups of T cell clusters depicted *via* a UMAP map. **(E)** Displaying the beginnings and endings of pseudo-time trajectories. The colors from dark to light represent the order of pseudo-time. **(F)** Pseudo-time analysis revealing the transition from CD4^+^ T conventional cells and CD8^+^ T naive cells to CD8^+^ T effector cells and CD4^+^ T regulatory cells. **(G–I)** GO, KEGG enrichment analysis and GSEA indicating the potential function of T cell-related genes.

Our cell-cell interaction analysis indicated that in terms of interaction numbers and weights, T cells had the strongest correlation with other cell types (Figures 7A,B). We identified several intercellular signaling pathways in seven key epithelial cell clusters (Figure 7C). Based on the analyses of four significant pathways consisting of COLLAGEN, MHC-I, APP and MIF signaling pathways (Supplementary Figures S1C–F), we delineated that T cells exerted predominant impact on the alteration of TME and needed in-depth investigation. In order to further define the potential role of T cells in the tumorigenesis of PCa, we extracted a subset of T cells and further subdivided them into four subtypes including CD4^+^ conventional T cells (CCR7), CD4^+^ regulatory T cells (FOXP3), CD8^+^ naïve T cells (LAG3), and CD8^+^ effector T cells (GZMA) based on conventional cell markers (Figure 7D) (Guo et al., 2018; Bian et al., 2024; Tuong et al., 2021). We modeled the developmental trajectory of cells by conducting pseudotime analysis. Cell trajectory profiles showed that T cells underwent evolutionary development (Figure 7E). We observed that CD4^+^ conventional T cells and CD8^+^ naïve T cells were gradually transformed into CD4^+^ regulatory T cells and CD8^+^ effector T cells (Figure 7F).

To explore the biological function of T cells in patients with PCa, we conducted GO, KEGG and GSEA-based gene enrichment analyses. First, GO analysis revealed the role of the enriched genes in biological processes, cellular components and molecular functions, respectively. We discovered that T cells primarily played a role in the regulation of cell-cell adhesion, mononuclear cell differentiation, lymphocyte differentiation, positive regulation of cytokine production, leukocyte cell–cell adhesion, external side of plasma membrane, actin binding, GTPase regulator activity, and nucleoside-triphosphatase regulator activity (Figure 7G). Additionally, KEGG analysis indicated that biological processes, such as human T-cell leukemia virus 1 infection, Epstein-Barr virus infection, T cell receptor signaling pathway, natural killer cell-mediated cytotoxicity, human immunodeficiency virus 1 infection, and human cytomegalovirus infection were correlated with T cells (Figure 7H). Finally, we discovered that CIITA_TARGET_GENES, HSF2_TARGET_GENES, KLF3_TARGET_GENES and MORC2_TARGET_GENES pathways were downgraded in PCa, based on the GSEA results (Figure 7I).

## 4 Discussion

Due to the failures of post-prostatectomy and radiotherapy in curing recurrent and locally advanced PCa, it is reasonable to attach great importance to novel immunotherapy (Sokoloff et al., 2004). Having previously shown that checkpoint inhibitors could bring favorable healing efficacy for patients with PCa, we have now explored a signature set of CARMR genes for anticipating the prognosis and drug response of patients with PCa in this report (Vietri et al., 2021; Pritchard et al., 2016). Additionally, cell death may maintain the balance through removing tumor cells, and this cell function was defective in metastatic and castration-resistant advanced PCa (Campbell and Leung, 2021; Zhu M. et al., 2023). Cuproptosis, as a newly discovered form of cell death, differs from other cell death modalities in its reliance on mitochondrial respiration (Tsvetkov et al., 2022). Meanwhile, growing evidence suggests that RNA-based epigenetic pathways are dysregulated in human diseases and may be ideal targets for cancer treatment (Barbieri and Kouzarides, 2020). In this research scenario, we investigated the potential connection between CARMRs and PCa.

First, we conducted univariate Cox regression and Lasso regression analyses to screen for RFS-related CARMRs and established a model to better predict prognosis and guide the stratified treatment of patients with PCa. An ROC curve was generated to validate the robust prognostic accuracy of the model. Ultimately, six CARMRs (ALYREF, DNMT1, DNMT3B, EXO1, HNRNPA2B1, TRDMT1) were included in our analysis to establish a prognostic model for estimating the risk of PCa recurrence. By reviewing previous studies, we discovered that prognostic genes played a key role in the tumorigenesis and advancement of PCa. Initially, ALYREF is determined to function as a reader of the 5-methylcytosine modification, playing a crucial role in stabilizing the associated mRNA and modulating its expression at the post-transcription level, thereby involving in cellular metabolism and movement (Zhang et al., 2021; Zhao et al., 2024b; Nulali et al., 2024). Building on a prior study, we propose a hypothesis that ALYREF may interact with the 5-methylcytosine modification on ACC1 mRNA and trigger the proliferation and lipid synthesis of PCa cells through activating the CDK13/NSUN5/ACC1 pathway (Zhang Y. et al., 2023). The DNMT3 gene family includes DNMT3A and DNMT3B, which are capable of methylating CpG sites and stably maintaining methylation patterns (Chen et al., 2003). By interacting with PI3K-Akt signaling pathway, DNMT3B and DNMT1 effectively silence the expression of tumor suppressor gene by increasing methylation levels and facilitate the malignant transformation of PCa cells (Agarwal et al., 2013; Zhu et al., 2021). EOX1 regulates the reprogramming of lipid metabolism by suppressing the P53 signaling pathway and promotes the progression of PCa (Wang et al., 2024). Moreover, distinct from other CARMRs, HNRNPA2B1 assumes a pivotal role in N6-methyladenosine methylation to regulate the TGF-β and FOXO pathways, influencing the phenotype of PCa and its response to conventional treatment (Qi et al., 2023; Liyanage et al., 2024). Consequently, CARMRs appear to modulate metabolism by amplifying the function of specific mRNAs, thereby modifying the phenotype of PCa. The summary of biological functions of the CARMRs were listed in Table 3.

**TABLE 3:** The biological functions and potential roles of CARMRs.

Gene name	Main function	Signaling pathways	Role in prostate cancer progression
ALYREF	Involved in mRNA export from the nucleus	CDK13/NSUN5/ACC1 pathway	inducing
DNMT1	Maintains DNA methylation patterns during DNA replication	PI3K-Akt signaling pathway	inducing
DNMT3B	Responsible for *de novo* DNA methylation	PI3K-Akt signaling pathway	inducing
EXO1	Involved in DNA repair, particularly in the 5′ to 3′ exonuclease activity	P53 signaling pathway	inducing
HNRNPA2B1	Involving in mRNA processing and transport	TGF-β and FOXO pathways	inducing
TRDMT1	Catalyzes the methylation of tRNAs	—	inducing

We carried out univariate Cox regression analysis to explore the role of clinicopathological parameters in the prognosis of PCa and our results showed the prognostic value of age, Gleason score, and pathological T stage and a correlation with risk score. We integrated all the factors to ascertain the status and advancement of this disease. We employed gene functional enrichment analysis to identify the potential functions of CARMRs in PCa: our data showed that CARMRs were correlated with muscle organ development and muscle fiber movement. Previous studies have shown that ADT treatment for PCa may lead to muscle atrophy and weakness through reducing Ca^2+^-sensitivity in Type I and II muscle fibers (Lamboley et al., 2018). Conversely, the organism of PCa patients tended to add the abundance and contraction ability of skeletal muscle to positively regulate the TME (Rocha-Rodrigues et al., 2021). Except for the adaptive regulatory, muscle cells produced more interleukins 4 and 13 for the growth of cancer stem cells and their interaction was essential for the cancer cell fusion and the generation of drug-sensitive phenotype (Uygur et al., 2019). Moreover, other studies have shown that cardiac and skeletal muscle mass is reduced in the absence of anti-cancer treatment (Baumfalk et al., 2019; Rollins et al., 2017). In this case, it is possible that tumor cells fuse together, inducing higher cytokine production from adjacent skeletal muscle cells and generate a metastatic phenotype by increasing myosin content. Besides, cardiac and skeletal muscle cells in other regions were consumed accordingly. Our point of view may be validated if there is evidence that the myosin expression profile in PCa influences the formation of a metastatic phenotype (Makowska et al., 2015). The evaluation of chemotherapy drugs indicated that doxorubicin and docetaxel were more effective when applied to patients with a high-risk of PCa recurrence. Studies have also demonstrated that doxorubicin and docetaxel exhibit anti-tumor activity in metastatic PCa (Petrioli et al., 2008; Fizazi et al., 2022).

It is well-established that immune responses play a dominant role in tumor development and anti-tumor therapy. To guide immunotherapy of patients with PCa, we investigated the immune infiltration in PCa tissue (Hawley et al., 2023). As key components of TME, immune cells and stromal cells are significantly correlated with immune therapy and prognosis of PCa. From the results of our study, the high-risk group exhibited increased abundance of M0 macrophages, M2 macrophages, and Tregs, and a significant reduction in plasma cells and mast cells. M0 macrophages are a plastic cell population that can change their phenotype under the influence of environmental signals such as radiation injury, potentially transitioning to tumor-associated macrophages (TAMs) (Qiu et al., 2018). TAMs are known to impact tumor progression through cell proliferation, angiogenesis, adaptive immune control, and metastasis, making them an attractive therapeutic target in PCa immune therapy (Jairath et al., 2020). To be specific, circSMARCC1 could activate the miR-1322/CCL20/CCR6 signaling pathway and induce the proliferation of TAMs to impact tumorigenesis (Xie et al., 2022). M2 macrophages may be polarized to influence metastasis and excessive proliferation of PCa cells *via* the IL17/CTSK/EMT axis (Wu et al., 2022). Referring to previous studies, Tregs have been shown to inhibit TME in various cancers, and to induce bone metastasis in PCa, which portends a poor prognosis (Liu et al., 2023; Meng et al., 2021; Alvisi et al., 2022; Boucher et al., 2023). Tregs are known to utilize the GIT/PAK/PIX complex to downgrade the anti-tumor response (Pedros et al., 2017). Clinical analysis has also corroborated the positive correlation between patient mortality rate and the degree of M2 macrophage and Tregs infiltration (Erlandsson et al., 2019). In conclusion, we reasoned that the study of TME may provide new ideas for regulating the immune status of tumor tissues, inhibiting tumor growth, and achieving a better prognosis.

To some extent, IC_50_ values had the capacity to characterize the natural response of PCa cells to chemotherapy agents (Xu et al., 2022). Our drug sensitivity analysis showed that doxorubicin and docetaxel may achieve better healing efficacy when applied to high-risk PCa subgroups. In an animal experiment, docetaxel was confirmed to remodel TME and enhance lymphocyte infiltration through activating the cGAS/STING pathway in PCa (Ma et al., 2022). Additionally, by conducting clinical trials, we found that doxorubicin and docetaxel combined with epirubicin demonstrated favorable efficacy in patients with advanced hormone-refractory PCa (Petrioli et al., 2008; Tannock et al., 2004; Petrylak et al., 2004). ALYRFE, functioning as a binding protein, can participate in diverse regulatory mechanisms, consisting of pre-mRNA processing, mRNA stability and mRNA methylation and facilitate the emergence of malignant phenotypes and the development of drug resistance (Zhao Y. et al., 2024; Zhong et al., 2024). Furthermore, across various tumor types, it has been observed that the increased transformation of ALYRFE into the nucleus leads to the elevated levels of 5-methylcytosine methylation, thereby promoting the drug resistance through the activation of a distinct molecular pathways (Shi et al., 2025; Huang et al., 2025; Xu et al., 2020). Similarly, TRDMT1 is capable of methylate both tRNA and mRNA, thereby promoting the stability of RNA and enhancing protein synthesis (Lewinska et al., 2023). An increase in expression of TRDMT1 and corresponding methylation levels results in the heightened resistance observed in multiple cancer cells (Lewińska et al., 2022; Lai et al., 2025). Conclusively, the CARMRs contribute to the therapeutic failure through enhancing the methylation levels. Furthermore, these findings may offer novel perspectives for addressing resistance issues in advanced PCa, potentially leading to the development of more effective treatment strategies.

We explored the relationship between prognostic genes and TME at the single-cell level. Our findings showed that most of the enriched genes were expressed in epithelial cells and T cells, which constitute the majority of tumor tissue and play an important role in the progression of PCa. The analysis of cell-cell interactions illustrated the strong interactions between T cells and others, which indicated that the heterogeneity of T cells could guide immunotherapy and determine patient prognosis. We subdivided T cells and modeled the developmental trajectory of T cells. CD8^+^ T effector cells and CD4^+^ T regulatory cells evolved from CD4^+^ T conventional cells and CD8^+^ T naïve cells. Therefore, we hypothesized that the expression of prognostic genes may promote the transition to CD4^+^ T regulatory cells and contribute to the poor prognosis. According to the results of our functional enrichment analysis, prognostic genes may be crucial in the differentiation of immune cells. Referring to other studies, we acknowledge that immune cell differentiation may cause immunosuppressive phenotypes. When monocytes transform to dendritic cells *via* tumor stroma-derived factors, the expression of CD14 and PD-L1 may elevate and hinder the destruction of immune cells in PCa (Spary et al., 2014). Alternatively, inhibiting the differentiation of MDSC and enhancing the proliferation of T cells may reverse its immune phenotype (Peng et al., 2022). Further, we noticed that target genes comprising CIITA, HSF2, KLF3 and MORC2 are strongly linked with RNA methylation (Mishra et al., 2010; Zhu J. et al., 2023; Tan et al., 2023; Chen F. et al., 2021). Of note, HSF2 impacts cell-cell adhesion and is positively correlated with a favorable prognosis (Björk et al., 2016). These observations are consistent with our GSEA results. Our current findings show that CARMRs may reshape TME by affecting the differentiation of immune cells. Eventually, this leads to enhancement of an invasive phenotype in PCa.

However, our study has some limitations. First, our analysis was based on a secondary analysis of public database data. These retrospective data were subject to selection biases, and this may have affected the accuracy of our analytical results. Additionally, there was a lack of a sufficient number of PCa samples to validate the applicability of the model, and the specific mechanisms by which the model genes may regulate PCa development remain elusive. Selection and sample biases may have been generated in our study since the clinical samples were selected from variously sourced datasets. Therefore, further *in vitro* and *in vivo* experimentation is needed to validate our results. Utilizing cell line experimentation, we would be able to compare the expression of prognostic genes of different invasive capacities in PCa cells to support our results.

## 5 Conclusion

To summarize, we have identified a correlation between RNA methylation and cuproptosis and were able to select six CARMRs to construct a risk stratification model for patients with PCa. Additionally, the relationship between TME and risk subgroups was analyzed by integrating single cell and bulk sequencing data to enable individualized immunotherapy. Through an in-depth investigation, we believe that our study has revealed a potential mechanism of PCa tumorigenesis that will support a higher efficacy therapeutic program.

## Data Availability

The original contributions presented in the study are included in the article/Supplementary Material, further inquiries can be directed to the corresponding authors.

## Supplementary material

SUPPLEMENTARY FIGURE S1 Integrating single-cell data of PCa cells, intercellular communication analysis and the alteration of genes in pseudotime analysis. **(A-B)** The single-cell atlas of PCa before and after harmony processes. **(C-F)** Heatmaps depicting the network centrality scores and potential roles of the top four signaling. **(G)** Heatmaps manifesting the expression of differentially expressed genes (DEGs) with the timing changes.
