# Supplementary figures and images for: Exploring the impact of cuproptosis on prostate cancer prognosis via RNA methylation regulation based on single cell and bulk RNA sequencing data
Source: Front Pharmacol. 2025 Apr 1;16:1573611. doi: 10.3389/fphar.2025.1573611 (PMC11996820; doi:10.3389/fphar.2025.1573611)

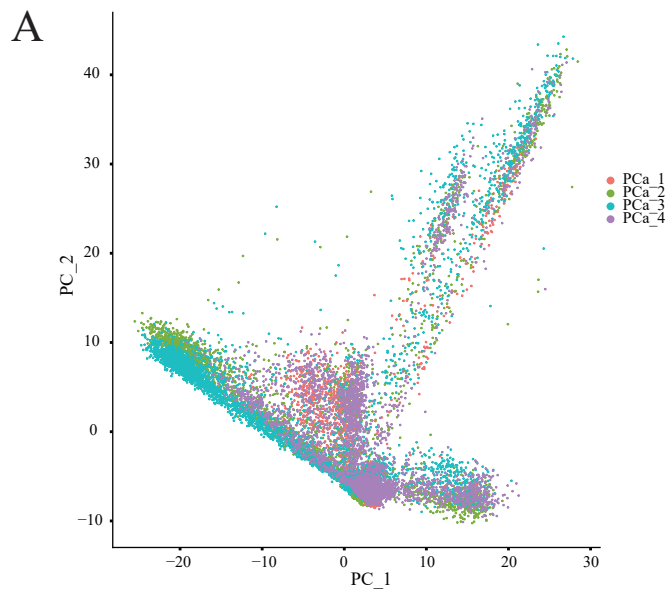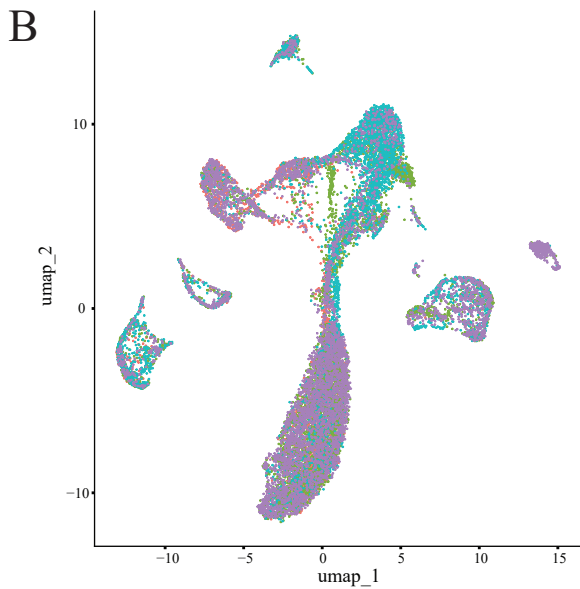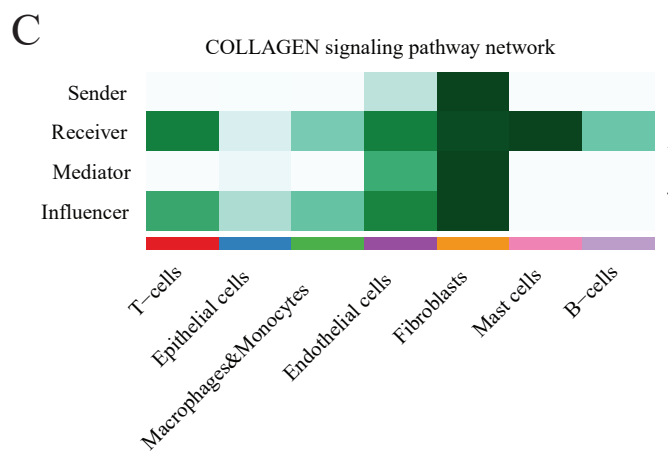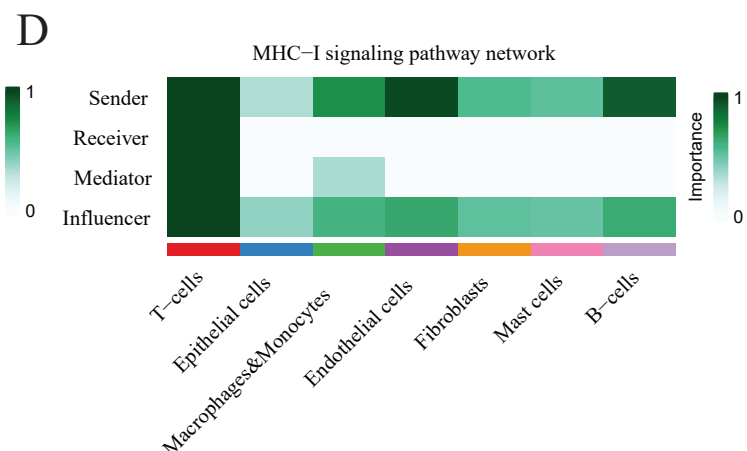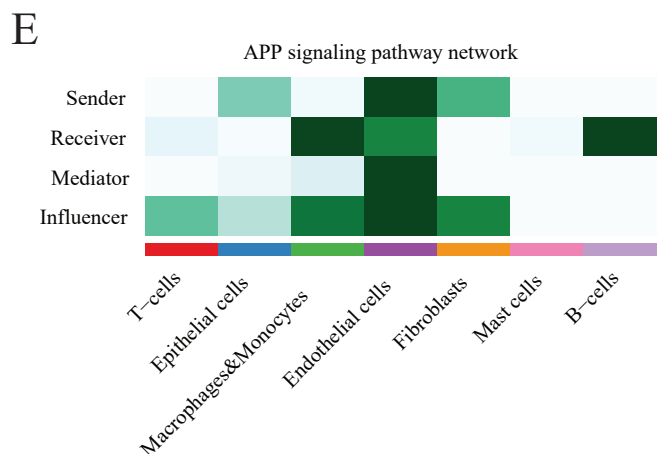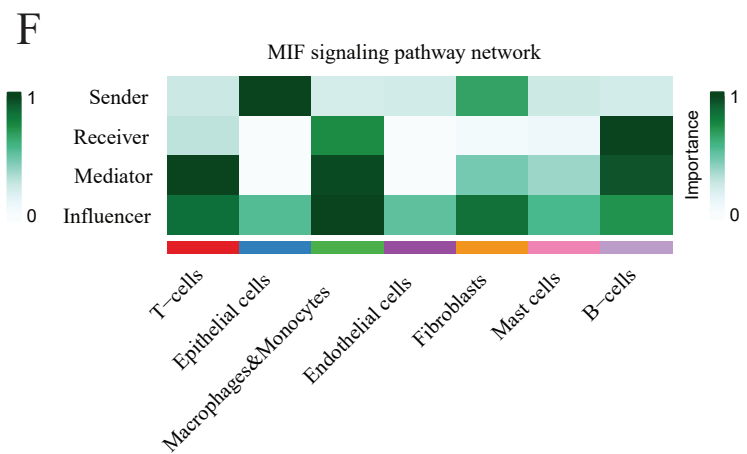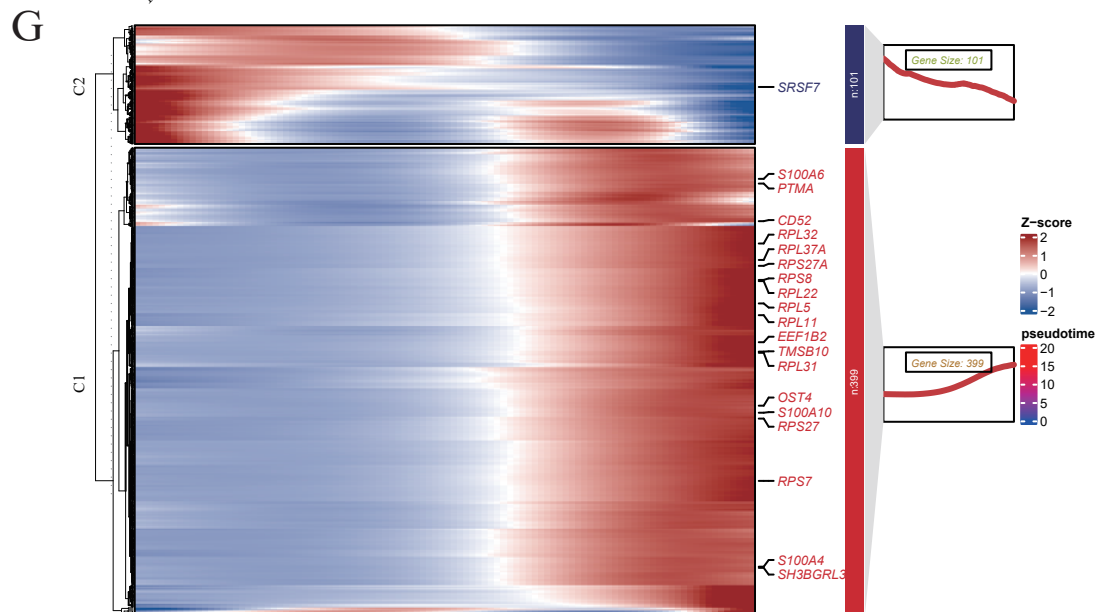

Supplement: Supplementary file 1 [file DataSheet1.PDF]
